# Blebbistatin reveals beneficial effects on the cystometric parameters in an animal model of detrusor overactivity

**DOI:** 10.1007/s00210-019-01640-3

**Published:** 2019-03-09

**Authors:** Andrzej Wróbel, Łukasz Nowakowski, Urszula Doboszewska, Ewa Rechberger, Małgorzata Bańczerowska-Górska, Edyta Wlaźlak, Izabela Zakrocka, Piotr Wlaź, Andrzej Semczuk, Jarosław Dudka, Ewa Poleszak

**Affiliations:** 1 Second Department of Gynecology, Medical University of Lublin, Jaczewskiego 8, 20-954 Lublin, Poland; 2 Department of Animal Physiology, Institute of Biology and Biochemistry, Faculty of Biology and Biotechnology, Maria Curie-Sklodowska University, Akademicka 19, 20-033 Lublin, Poland; 3 Gynaecological and Obstetrics Hospital in Wałbrzych, Paderewskiego 10, 58-301 Wałbrzych, Poland; 4 Clinic of Operative Gynecology and Gynecologic Oncology, 1st Department of Gynecology and Obstetrics, Medical University of Lodz, Wileńska 37, Łódź, 94-029 Poland; 5 Chair and Department of Nephrology, Medical University of Lublin, Jaczewskiego 8, 20-954 Lublin, Poland; 6 Chair and Department of Toxicology, Medical University of Lublin, Chodźki 8, 20-093 Lublin, Poland; 7 Chair and Department of Applied Pharmacy, Medical University of Lublin, Chodźki 1, 20-093 Lublin, Poland

**Keywords:** Blebbistatin, OAB, Detrusor overactivity, Rats, Cystometry

## Abstract

The aims of the study were to determine the effectiveness of blebbistatin (BLEB) on detrusor overactivity (DO) in an animal model induced by retinyl acetate (RA) and, because of potential urothelial permeability, to evaluate the degenerative impact of BLEB on the urothelium. Three days after RA instillation into the urinary bladder, BLEB was administered into the bladder and immediately after cystometric assessment was performed. Furthermore, Evans Blue extravasation into bladder tissue and urothelium thickness were measured. Sixty female Wistar rats were used and randomly assigned to one of four groups (*n* = 15 in each group): (1) control, (2) RA, (3) BLEB, and (4) RA + BLEB. RA administration induced changes in cystometric parameters reflecting DO, as previously reported. Treatment with BLEB did not significantly alter cystometric parameters in rats which did not receive RA. Administration of BLEB to rats pretreated with RA reversed changes in cystometric parameters induced by RA in basal pressure, threshold pressure, detrusor overactivity index, amplitude of nonvoiding contractions, frequency of nonvoiding contractions, voided volume, volume threshold, intercontraction interval, bladder compliance, and volume threshold to elicit nonvoiding contractions. There were no significant differences in Evans Blue extravasation into bladder tissue or urothelium thickness between the groups. The current research provides new data on the possible utility of blebbistatin in the pharmacotherapy of DO, which is an important feature of overactive bladder (OAB). Further studies in human patients with DO/OAB are warranted to confirm these preclinical results.

## Introduction

The vast majority of patients treated for overactive bladder syndrome (OAB) display urodynamically confirmed detrusor (bladder smooth muscle) overactivity (DO) and are administered antimuscarinic (anticholinergic) drugs, the first-line therapy for this condition (Abrams et al. 2003). Antimuscarinics are effective in reducing urgency and frequency. However, it has been estimated that 4–31% of all patients discontinue treatment within 12 weeks due to the side effects or lack of efficacy, or a combination of both (Sexton et al. 2011). In a long-term assessment, up to 50% of all patients discontinue the intake of the selective antimuscarinic drugs (Leron et al. 2018).

β_3_-adrenergic receptor agonists or onabotulinum injections are firmly established alternatives for the anticholinergic drugs and possess good efficacy, but not in treating the complete spectrum of OAB symptoms (Thiagamoorthy et al. 2016). Thus, studies aimed at searching for alternative OAB treatment methods are carried out. In addition to novel mechanism of action, local (intravesical) drug administration may be beneficial because it enables avoiding systemic side effects and preserving clinical effectiveness; however, low permeability of the urothelial barrier is a limitation of such a drug delivery system (Zacche et al. 2015).

It has been suggested that inhibition of class II myosins is a potential strategy for reducing bladder muscle contractions (Nowakowski et al. 2012). Myosins are a superfamily of motor proteins which are major regulators of contractile properties of smooth, cardiac, and skeletal muscles (Reiser 2018). 24 classes (I–XXIV) of myosins have been described (Foth et al. 2006). Class II consists of skeletal, cardiac, smooth, and nonmuscle subclasses (Sellers 2000). Smooth muscle myosin comprises one pair of myosin heavy chains and two pairs of myosin light chains (Adelstein and Eisenberg 1980). Myosin heavy chain exists in four isoforms which differ both at the carboxyl terminus (SM1 and SM2 isoforms) and at the amino terminus (SM-A and SM-B isoforms) (Andersson and Arner 2004; Babu et al. 2000). The ratio of SM1 to SM2 is tissue-specific (Babu et al. 2000). In the adult rat urinary bladder, the relative content of SM1 is 70% of the myosin heavy chain (Andersson and Arner 2004). Furthermore, both SM1 and SM2 isoforms can contain an insert enabling four possible isoforms: SM1-A, SM1-B, SM2-A, and SM2-B. Urinary bladder tissue has high expression of the inserted myosin isoform with 80–90% SM-B at the mRNA level. The relative myosin light chain isoform LC17b is low in the urinary bladder tissue (10% in rat (Andersson and Arner 2004)). An inverse relationship between the maximal shortening velocity of a muscle and the relative content of myosin light chain isoform LC17b has been demonstrated (Malmqvist and Arner 1991). In rabbits, it was found that the bladder of control animals displayed almost 100% of LC17a, while the bladder of animals with bladder outlet obstruction displayed an increased level of LC17b, which was associated with decreased maximum velocity of shortening (DiSanto et al. 2003). The expression of nonmuscle myosins is also low in adult urinary bladder, i.e., 10% of total heavy chain in rats (Andersson and Arner 2004).

Blebbistatin (BLEB) has been discovered with the aid of a high-throughput molecule screen for inhibitors of nonmuscle myosin II (Straight et al. 2003). Furthermore, it was demonstrated that BLEB inhibits both nonmuscle and muscle myosins (Newell-Litwa et al. 2015). Moreover, it was demonstrated that BLEB inhibiting potential is higher toward the SM-B (which is present in higher amounts in the urinary bladder (Andersson and Arner 2004)) compared to the SM-A type (Rhee et al. 2006) (which is found in lower abundance in the bladder (Andersson and Arner 2004)). On the contrary, Eddinger et al. (2007) have found that chicken gizzard tissue with dominating SM-B subtype was less susceptible to BLEB than that consisting of SM-A carotid artery.

An important fact is that BLEB relaxed both rat and human bladder smooth muscles in vitro and it significantly altered urodynamic parameters in vivo to values corresponding to decreased bladder overactivity (Zhang et al. 2011a). The study of Zhang et al. (2011a) was conducted on the “normal” bladder, without induced DO. In addition, intravesically administered BLEB was shown to relax bladder smooth muscles in a model of partial bladder outlet obstruction (PBOO) (Zhang et al. 2011b). Bladder outlet obstruction may lead to symptoms of OAB (Dmochowski and Gomelsky 2009), but not all patients with OAB have bladder outlet obstruction (Al-Zahrani and Gajewski 2016). Therefore, we aimed at assessing the effects of BLEB in animal models of DO which are not associated with bladder outlet obstruction, i.e., in models induced by 13-*cis*-retinoic acid or retinyl acetate administration. We have previously demonstrated that a 1-week treatment with BLEB administered as an intra-arterial bolus attenuated changes in cystometric parameters induced by 13-*cis*-retinoic acid in female rats (Wróbel et al. 2019). To further explore the possibility of utilizing BLEB in the management of DO, here we assessed the effects of intravesical delivery of BLEB in a model induced by retinyl acetate instillation to the bladder.

## Materials and methods

### Drugs

The following drugs were used:

- Retinyl acetate (RA) (Sigma-Aldrich, Poznań, Poland) was diluted to 0.75% solution with a mixture of polysorbate 80 and saline.
- (±)-BLEB (Tocris, Bristol, England, UK): (±)-1,2,3,3a-Tetrahydro-3a-hydroxy-6-methyl-1-phenyl-4*H*-pyrrolo[2,3-*b*]quinolin-4-one, a small molecule, cell permeable, and selective inhibitor of the myosin-ADP-P_i_ complex blocking the myosin II in an actin-detached state (Kovacs et al. 2004), was dissolved in DMSO to a concentration of 125 nM.

The doses of the administered agents were based on literature data and were confirmed/adjusted in our laboratory in preliminary experiments (Wróbel and Rechberger 2017a; Zhang et al. 2011a).

### Animals

The study was performed following the European Communities Council Directive of 22 September 2010 (2010/63/EU). The experimental procedures and protocols were approved by the Local Ethics Committee (Lublin, Poland). The experiments were carried out on female Wistar rats, at the age of 4 weeks, initially weighing 200–225 g. The rats were derived from the Center of Experimental Medicine at the Medical University of Lublin. Animals were separately located in the metabolic cages (3700M071, Tecniplast, West Chester, PA, USA) with ad libitum access to food and water.

**Fig. 1:**
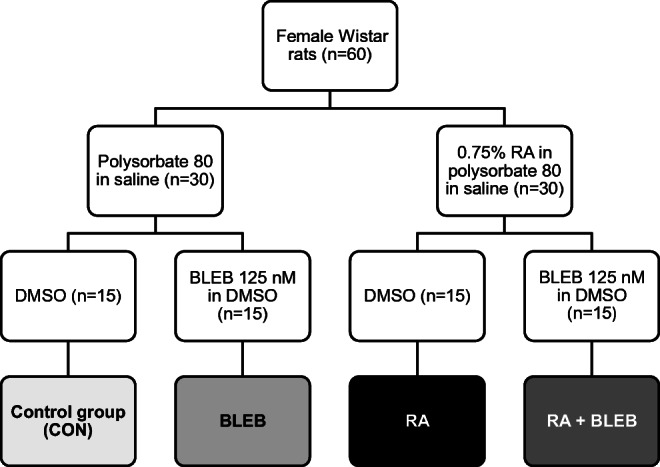
Experimental schedule. 60 female rats were randomly divided into groups (*n* = 30) that received intravesical instillation of RA (0.75% solution in polysorbate 80 in saline) or polysorbate 80 in saline. 3 days after, each group was subdivided into groups (*n* = 15) that received BLEB intravesically (at a concentration of 125 nM in DMSO) or DMSO, and immediately after cystometric assessment was performed. BLEB, blebbistatin; RA, retinyl acetate; CON, control group

A total of 60 female Wistar rats were used in the study. The rats were randomly divided into groups (*n* = 30) that received intravesical instillation of RA (0.75% solution in polysorbate 80 in saline) or polysorbate 80 in saline. Three days after, each group was subdivided into groups (*n* = 15) that received BLEB intravesically (at a concentration of 125 nM in DMSO) or DMSO, and immediately after cystometric assessment was performed (Fig. 1).

### Surgical procedures and RA administration

The surgical procedures have been previously described in detail (Wróbel and Rechberger 2017a; Wróbel and Rechberger 2017b). In brief, the first procedure was a bladder catheterization with a polyethylene catheter through the external urethral meatus. The bladder was emptied and the 0.75% RA solution or vehicle (polysorbate 80 in saline) was installed into the bladder using the previously inserted catheter until the intravesical pressure extended to 10 cm H_2_O. Five minutes later, the dilutions were removed and the bladder was washed mildly three times with 0.9% NaCl. The urethral catheter was removed and the 10-mm abdominal midline vertical incision was performed. Subsequently, a double lumen catheter was introduced through the fundus to the urinary bladder and fixed with the 6–0 suture. Abdominal wall was then sutured and closed in layers. The surgeries were implemented under the anesthesia attained by the intraperitoneal (i.p.) injection of 75 mg/kg of ketamine hydrochloride (Ketanest, Pfizer, Karlsruhe, Germany) and 15 mg/kg of xylazine (Sedazin, Biowet, Puławy, Poland). The choice of the type of anesthesia was made due to the reports stating that ketamine in combination with xylazine does not block the micturition reflex in female rats (Cannon and Damaser 2001).

### BLEB administration and conscious cystometry

Cystometric evaluation was performed 3 days after surgical procedures in conscious unrestrained animals. The bladder catheter was connected to a microinjection pump (CMA 100, CMA Microdialysis AB, Kista, Sweden) and to a pressure transducer (FT03, Grass Technologies, West Warwick, RI, USA) using a three-way stopcock. The rat bladder was infused with 125 nM of the BLEB or vehicle (DMSO) for 30 min. Conscious cystometry was then performed by slowly filling the bladder with physiological saline at a constant rate 0.05 ml/min to provoke repetitive voiding (Wróbel and Rechberger 2017a; Wróbel and Rechberger 2017b). The following cystometric parameters were recorded: basal pressure (BP, cm H_2_O), threshold pressure (TP, cm H_2_O), micturition voiding pressure (MVP, cm H_2_O), voided volume (VV, ml), post-void residual (PVR, ml), volume threshold (VT, ml), voiding efficiency (VE, %), intercontraction interval (ICI, s), bladder contraction duration (BCD, s), relaxation time (RT, s), bladder compliance (BC, ml/cm H_2_O), detrusor overactivity index (DOI, cm H_2_O/ml), frequency of nonvoiding contractions (FNVC, times/filling phase), amplitude of nonvoiding contractions (ANVC, cm H_2_O), and volume threshold to elicit nonvoiding contractions (VTNVC, %). The meaning of the measured parameters has been provided below.

Basal pressure is the lowest bladder pressure during the filling phase. In animals with zero residual volume, basal pressure is the passive pressure in an empty bladder, while in animals with residual volume, it corresponds to the pressure at this volume. Threshold pressure is the bladder pressure immediately before the onset of micturition contraction. Micturition voiding pressure is the maximum bladder pressure during micturition. As the rats do not seem to strain and have an abdominal pressure close to 0, micturition pressure is almost identical to the detrusor pressure. Voided volume is the volume of the expelled urine. Post-void residual is the volume of fluid remaining in the bladder at the end of micturition and is calculated as bladder capacity minus voided volume. Volume threshold has been calculated as the sum of voided volume and residual volume. Voiding efficiency has been calculated using the formula: [(voided volume/volume threshold) × 100]. Intercontraction interval is the interval between micturition voiding pressure and the next micturition voiding pressure. Bladder contraction duration is the interval between threshold pressure and basal pressure. Relaxation time is the interval between micturition voiding pressure and basal pressure. Bladder compliance has been calculated as the bladder capacity divided by the difference in the threshold pressure and baseline pressure, using the formula: [(VV + PVR)/(TP − BP)]. Bladder compliance is routinely measured as an index of bladder storage function. Since the bladder can be extended at a lower intrabladder pressure, higher compliance indicates a better storage function. Detrusor overactivity index is depicted as the quotient of the sum of amplitudes of all detrusor contractions during the filling phase and functional bladder capacity. Nonvoiding contraction is an increase in bladder pressure without a release of fluid from the urethra. Nonvoiding contractions higher than 2 cm H_2_O were used as a surrogate for detrusor overactivity. A voiding contraction was identified as a large increase in bladder pressure accompanied by the release of fluid from the urethra. Volume threshold to elicit nonvoiding contractions is the percent of total bladder filling volume and is a preclinical equivalent of the volume at the first involuntary detrusor contraction, which is measured during urodynamic investigations in humans.

After the cystometric assessment, bladder edema and urothelium thickness were measured.

### Bladder edema measurement

Bladder edema measurement has been previously described in detail (Wróbel et al. 2017). The results are presented as nanograms of Evans Blue per milligram of the bladder.

### Urothelium thickness measurement

The image analyzer computer system Leica Qwin 500 Image Analyzer (Leica Imaging Systems Ltd., Cambridge, England, UK) was used to evaluate the urothelium thickness in micrometers. A mean of 15 readings was estimated from five serial sections from the slides of each animal in each group using low magnification (× 10).

### Statistical analysis

The data were evaluated by the one-way analysis of variance (ANOVA) followed by Tukey’s post hoc test (Prism ver. 5.03, GraphPad Software, San Diego, CA, USA). The obtained results are presented as the mean ± S.E.M. *p* < 0.05 was considered as statistically significant with 95% confidence.

## Results

### The effects of BLEB treatment on RA-induced changes in cystometric parameters

Instillation of RA into the urinary bladder produced changes in cystometric parameters associated with DO. Increases in basal pressure, threshold pressure, detrusor overactivity index, amplitude of nonvoiding contractions, and frequency of nonvoiding contractions were observed in rats that received RA compared to the control group, while decreases were noted in voided volume, volume threshold, intercontraction interval, bladder compliance, and volume threshold to elicit NVC. Treatment with RA did not lead to significant changes in micturition voiding pressure, post-void residual, voiding efficiency, bladder contraction duration, or relaxation time.

**Fig. 2:**
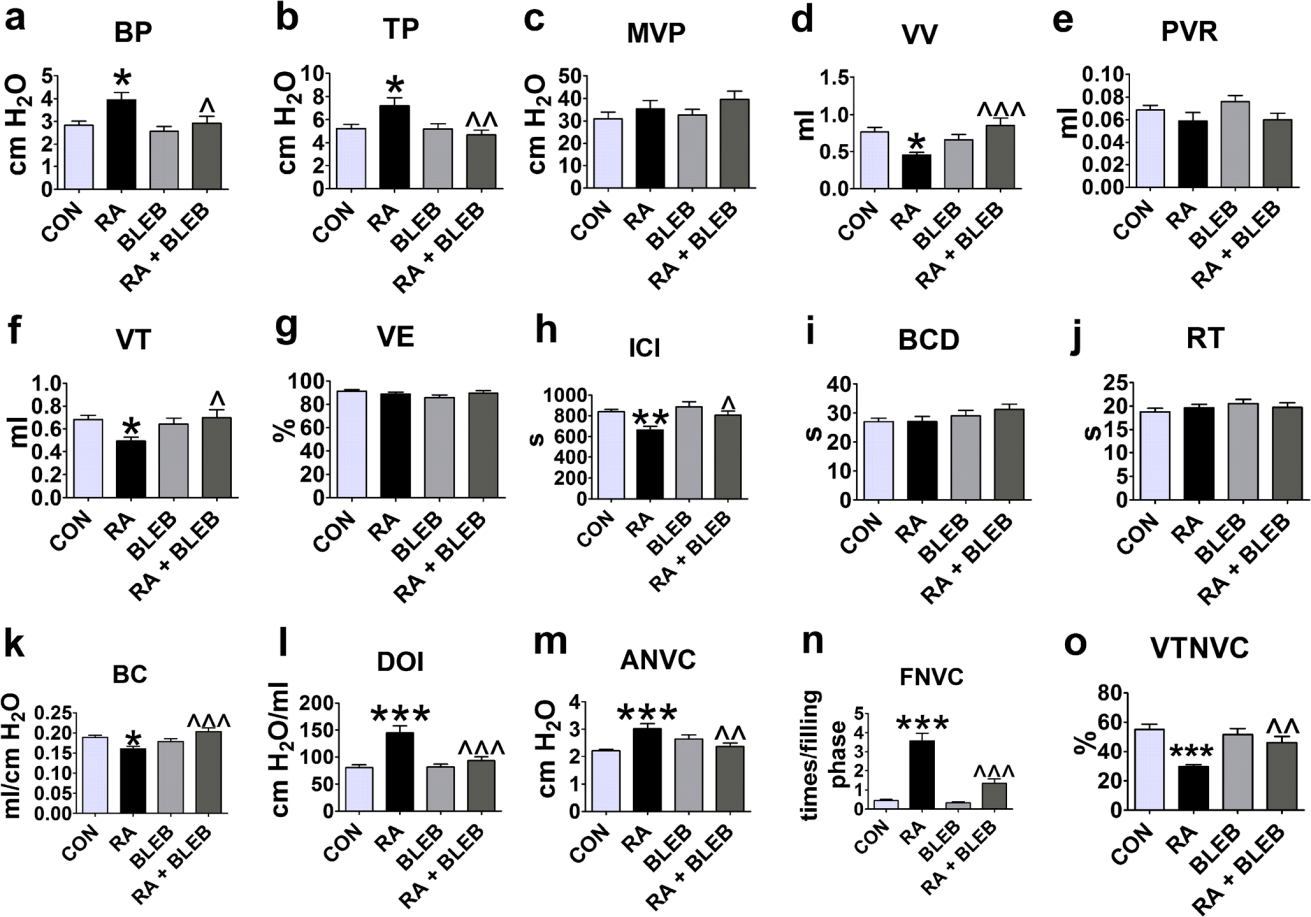
**a**–**o** The effects of BLEB on RA-induced DO. The rats received intravesical instillation of RA (0.75% solution in polysorbate 80 in saline) or vehicle (polysorbate 80 in saline). 3 days after, the rats received intravesically BLEB (at a concentration of 125 nM in DMSO) or DMSO, and immediately after cystometric assessment was performed. BLEB, blebbistatin; RA, retinyl acetate; CON, control group; DO, detrusor overactivity; BP, basal pressure; TP, threshold pressure; MVP, micturition voiding pressure; VV, voided volume; PVR, post-void residual; VT, volume threshold; VE, voiding efficiency; ICI, intercontraction interval; BCD, bladder contraction duration; RT, relaxation time; BC, bladder compliance; DOI, detrusor overactivity index; ANVC, amplitude of nonvoiding contractions; FNVC, frequency of nonvoiding contractions; VTNVC, volume threshold to elicit nonvoiding contractions. Data were analyzed by one-way ANOVA followed by Tukey’s post hoc test. Data are presented as the mean + SEM, *n* = 15 in each group. One-way ANOVA: BP (*F*(3, 56) = 5.378, *p* = 0.0025), TP (*F*(3, 56) = 5.092, *p* = 0.0035), MVP (*F*(3, 56) = 1.350, *p* = 0.2676), VV (*F*(3, 56) = 6.076, *p* = 0.0012), PVR (*F*(3, 56) = 1.899, *p* = 0.1403), VT (*F*(3, 56) = 3.494, *p* = 0.0214), VE (*F*(3, 56) = 1552, *p* = 0,2113), ICI (*F*(3, 56) = 6.492, *p* = 0.0008), BCD (*F*(3, 56) = 1.398, *p* = 0.2530), RT (*F*(3, 56) = 0.6753, *p* = 0.5708), BC (*F*(3, 56) = 6.434, *p* = 0.0008), DOI (*F*(3, 56) = 13.90, *p* < 0.0001), ANVC (*F*(3, 56) = 6.538, *p* = 0.0007), FNVC (*F*(3, 56) = 40.41, *p* < 0.0001), VTNVC (*F*(3, 56) = 10.58, *p* < 0.0001). *p < 0.05, **p < 0.01, ***p < 0.01 vs. control group (CON); ^*p* < 0.05, ^^*p* < 0.01, ^^^*p* < 0.001 vs. RA by the Tukey’s post hoc test

Administration of BLEB to rats that received vehicle instead of RA did not cause significant differences in any of the cystometric parameters, compared to the control group. Administration of BLEB to rats pretreated with RA reversed changes induced by RA in basal pressure, threshold pressure, detrusor overactivity index, amplitude of nonvoiding contractions, frequency of nonvoiding contractions, voided volume, volume threshold, intercontraction interval, bladder compliance, and volume threshold to elicit NVC. After BLEB treatment in rats pretreated with RA, no significant changes were found in micturition voiding pressure, post-void residual, voiding efficiency, bladder contraction duration, or relaxation time (Fig. 2).

### The measurement of the Evans Blue extravasation and urothelium thickness

One-way ANOVA demonstrated no significant changes in Evans Blue extravasation into bladder tissue or urothelium thickness between the groups (Table 1).

**Table 1:** The influence of intravesical administration of retinyl acetate (RA) (0.75%) and blebbistatin (BLEB) (125 nM) on Evans Blue extravasation and urothelium thickness

Treatment	Evans Blue (ng/mg)	Urothelium thickness (μm)
CON	24.80 ± 1.9	57.27 ± 2.12
RA	26.40 ± 1.40	50.53 ± 2.26
BLEB	27.93 ± 1.35	48.00 ± 3.87
RA + BLEB	25.40 ± 1.35	51.73 ± 2.05

All results are presented as the means ± SEM (*n* = 15 rats per group). The obtained data were analyzed by the one-way analysis of variance (ANOVA) followed by Tukey’s post hoc test

*CON*, control group (the control group received the vehicle used for RA administration (polysorbate 80 in saline) instead of RA and the vehicle used for BLEB administration (DMSO) instead of BLEB)

## Discussion

The aim of the study was to evaluate the influence of intravesical BLEB administration on DO induced by RA instillation into the bladder and to assess their impact on the urothelium. The obtained results showed no influence of the investigated substance (BLEB) on the cystometric parameters in healthy rats. The concentration of BLEB of 125 nM did not affect the bladder capacity or other crucial urodynamic parameters in healthy rats. This finding suggests that a proper dose selection for a myosin inhibitor is crucial in order not to produce negative changes to the physiology of the bladder.

Furthermore, BLEB normalized changes in cystometric parameters induced by RA, which corresponds to attenuation of DO. The important findings are a decrease in the basal pressure, threshold pressure, detrusor overactivity index, amplitude of nonvoiding contractions, and frequency of nonvoiding contractions following BLEB instillation in the bladder of RA-pretreated rats, compared to RA-pretreated rats that received vehicle. Furthermore, a significant increase in the voided volume, volume threshold, intercontraction interval, bladder compliance, and volume threshold to elicit NVC was observed in BLEB-treated rats. Moreover, BLEB did not affect the micturition voiding pressure, post-void residual, voiding efficiency, bladder contraction duration, and relaxation time. The above findings indicate that BLEB improves urine storage with no impairment of the voiding function. The significant reduction of the detrusor overactivity index, amplitude of nonvoiding contractions, and frequency of nonvoiding contractions in animals with DO, following BLEB administration, may indicate no influence of this molecule on the afferent micturition mechanisms. Furthermore, no changes in the bladder contraction duration and relaxation time point to constant outflow resistance as the result of the lack of the relaxing impact on the urethra.

One of the most important outcomes of the present study is a significant decrease in the detrusor overactivity index value following BLEB administration in RA-pretreated rats. The height of the waves of the detrusor pressure (Pdet) curve during cystometry is analyzed in order to measure this parameter. This technique has been used in urodynamic studies in humans, and studies have shown that the increase in detrusor overactivity index correlates with the severity of DO (Abrams 2003). This parameter is currently considered to be a more precise indicator of the contractile activity of the detrusor muscle, compared to the bladder compliance, basal pressure, amplitude of nonvoiding contractions, or volume threshold to elicit NVC.

Another interesting outcome is the increase in the volume threshold to elicit nonvoiding contractions, which was observed following BLEB administration in rats pretreated with RA. This parameter may be considered as a preclinical counterpart of the volume threshold to elicit the first involuntary detrusor contraction, which is measured during cystometry in humans (Behr-Roussel et al. 2012). An increase in this parameter correlates with the reduction in the micturition episodes, and it seems to be a very reliable marker of the effectiveness of the overactive bladder pharmacotherapy.

Nonmuscle myosin II is among the BLEB targets. However, the role of this type of myosin has not yet been fully described. In a study on knockout mice, Morano et al. (2000) have found that the nonmuscle myosin component can develop a slow contraction of the smooth muscle in newborn animals. In another study, in research upon the bladder wall of hypertrophying mouse urinary bladders, Boberg et al. (2015) evaluated whether the protein kinase C (PKC) pathway activation is the appropriate animal model for bladder overactivity. They found that BLEB administration inhibits PKC-induced contractions of the bladder muscle. Thus, as BLEB acts on nonmuscle myosin, the authors suggest that this may be an important mechanism with regard to the detrusor function in the described animal model. Furthermore, activation and regulation of the nonmuscle myosin activity are not affected by the physiological muscarinic influence and might be an important, yet unknown, trigger point of the detrusor contraction (Boberg et al. 2015).

BLEB does not block the interaction between myosin and actin. Thus, filaments are able to interact and their movements are preserved. However, complete blockage of the filaments is possible if a higher concentration of the substance is used (Limouze et al. 2004). This property of BLEB makes it a potentially suitable substance for bladder treatment research. When compared to standard onabotulinum treatment, myosin II inhibitor does not completely block the activity of the bladder smooth muscle and, as a consequence, is potentially much safer for long-term treatment.

Our results are consistent with the findings described by Zhang et al. (2011a). In this well-designed study, the authors found that instillation of BLEB into the bladder increases its capacity and decreases micturition frequency. Zhang et al. (2011a) suggest that BLEB may be more efficient in reducing the contraction induced by the K^+^-channel openers, hence, targeting downstream of the Ca^2+^/calmodulin myosin phosphorylation signaling pathway, compared to the antimuscarinic treatment. However, the abovementioned study was conducted on the normal bladder without DO.

Here, we used animals in which DO was induced by a gently irritating agent—RA (Wróbel et al. 2015). RA belongs to the family of retinoids, whose chemical structures are related to vitamin A (Tang and Gudas 2011). We have previously shown that BLEB attenuated changes in the cystometric parameters in a model of DO induced by administration of another retinoid, 13-*cis*-retinoic acid, in rats (Wróbel et al. 2019). Retinoids activate transient receptor potential channel vanilloid (TRPV) subtype 1 (TRPV1), which leads to the stimulation of nociceptive sensory neurons and, consequently, to sensory hypersensitivity (Alique et al. 2006; El Andaloussi-Lilja et al. 2009; Yin et al. 2013). Studies of the lower urinary tract have indicated that TRPV channels, including TRPV1, TRPV2, TRPV4, TRPM8, and TRPA1, are expressed in the bladder and may act as sensors of stretch and/or chemical irritation (Andersson 2016). Furthermore, it was shown that Ca^2+^ influx through TRPV4 channels regulates the interaction between nonmuscle myosin and actin binding protein Flightless I (Arora et al. 2017). Taken together, it is plausible that the mechanism underlying DO development in models induced by the use of retinoids as well as the beneficial effects of BLEB in these models is mediated by their effects on TRPV channels.

Importantly, we did not observe the influence of BLEB upon the Evans Blue extravasation or on urothelium thickness. This finding may indicate that BLEB administration does not induce a degenerative process inside the bladder wall and, as a consequence, points to the safety of the drug in the in vivo studies.

We have neither assessed pharmacokinetics of BLEB, nor the prolonged effects of BLEB instillation. Thus, we cannot suggest whether it would be possible to treat by a single instillation of this drug. However, it is plausible that BLEB will exert long-lasting effects given the fact that a single basolateral amygdala complex treatment with BLEB produced a long-lasting disruption of context-induced drug seeking, which lasted at least 30 days (Young et al. 2016).

## Conclusions

The current research provides new data on the possible utility of BLEB in the pharmacotherapy of DO. Three main findings should be particularly underlined:

1. BLEB did not influence the cystometric parameters in healthy rats.
2. BLEB normalized changes in cystometric parameters (associated with DO) to values corresponding to decreased DO.
3. BLEB did not induce degenerative changes in the urothelium after local administration.

Further studies in human patients with DO/OAB are warranted to confirm these preclinical results.
